# Trend of Mortality Rate and Years of Life Lost due to Cerebrovascular Diseases in Fars Province, Iran (2004–2019)

**DOI:** 10.34172/aim.2023.30

**Published:** 2023-04-01

**Authors:** Habibollah Azarbakhsh, Najibullah Baeradeh, Seyed Parsa Dehghani, Jafar Hassanzadeh, Maryam Janfada, Ahmadreza Razeghi, Alireza Mirahmadizadeh

**Affiliations:** ^1^Student Research Committee, Shiraz University of Medical Sciences, Shiraz, Iran; ^2^School of Medicine, Shiraz University of Medical Sciences, Shiraz, Iran; ^3^Research Center for Health Sciences, Institute of Health, Department of Epidemiology, Shiraz University of Medical Sciences, Shiraz, Iran; ^4^Department of Statistics, Health Vice-chancellor, Shiraz University of Medical Sciences, Shiraz, Iran; ^5^Non-Communicable Diseases Research Center, Shiraz University of Medical Sciences, Shiraz, Iran

**Keywords:** Cerebrovascular diseases, Iran, Joinpoint regression, Mortality rate, Trend, Years of life lost

## Abstract

**Background:**

According to the Global Burden of Disease Study (GBD), cerebrovascular diseases are the second leading cause of death in the world. This is a cross-sectional study on deaths due to cerebrovascular diseases in southern Iran.

**Methods:**

In this cross-sectional study, data on all deaths caused by cerebrovascular diseases in the Fars province between 2004 and 2019 was extracted from the Electronic Death Registry System (EDRS). To eliminate or minimize the influence of age composition, standardized mortality rate was used based on the 2013 Segi standard populations of low- and middle-income countries. In order to measure the years of life lost (YLL) from cerebrovascular diseases, the standardized life table was considered. The Joinpoint Regression method was used to examine the trend of the crude and standardized mortality rate and the YLL rate.

**Results:**

Over the study period, 24,051 deaths occurred due to cerebrovascular diseases in Fars with 12,586 cases in men (52.3%). The trend of standardized mortality rate in males and females was decreasing (*P* value=0.001 and<0.001 for males and females, respectively). All YLL due to premature mortality from cerebrovascular disease during the 16-year study period were 119,436 (3.8 per 1000 persons) in men, and 111,172 (3.6 per 1000 persons) in women. Based on the joinpoint regression, the 16-year trend of YLL rate due to premature death was decreasing: annual percent change (APC) was -1.6% (95% CI -3.4 to 0.3, *P*=0.098) for males, and -2.0% (95% CI -3.6 to -0.4, *P*=0.017) for females.

**Conclusion:**

The trend of mortality rate and YLL caused by cerebrovascular diseases has decreased in our study. Necessary measures, mainly primary and secondary prevention, should be taken to continue the diminishing trend of cerebrovascular diseases.

## Introduction

 Cerebrovascular disease is a general term that includes various brain vascular disorders. Most deaths due to cerebrovascular disease are the result of stroke.^1^ The Global Burden of Disease Study estimates that cerebrovascular disease is the second leading cause of death in world, accounting for 9.5% of deaths in low- and middle-income countries and 9.9% of deaths in high-income countries.^2^ In 2015, the rate of dementia due to cerebrovascular disease in upper-middle-income countries was 120.9 in 100 000 people, while the global rate was 81.9 in 100 000 people.^3^ According to the World Health Organization (WHO), cardiovascular diseases caused 17.3 million deaths in 2008 (30% of all deaths worldwide), which will increase to 23.3 million in 2030. In Eastern Mediterranean countries such as Iran, being in an epidemiological transition, non-communicable diseases are responsible for 53% of all deaths.^4^ Today, cardiovascular and cerebral disease deaths are twice as high as the total deaths from HIV, tuberculosis, and malaria.^5^ Cerebrovascular disease mortality has declined for decades in most parts of the world but has remained very high in Russia and several parts of Central and Eastern Europe.^6^ In a study conducted in China, in rural areas of the country, 51% of life expectancy lost due to cardiovascular disease was due to cerebrovascular disease.^7^ Mortality is the most objective measure of health problems, and YLL are a major component of the disease burden, especially in low- and middle-income countries such as Iran.^8^ A literature review on stroke burden in developing countries and Asia lacks comprehensive information on Iran.^9^ Considering that no study has been done to date, the purpose of the study was to determine the trend of mortality rate and years of life lost (YLL) due to cerebrovascular diseases in the Fars province.

## Materials and Methods

 This cross-sectional study collected total deaths due to cerebrovascular diseases in the Fars province from the electronic population-based death registration system (EDRS). Fars is located in southern Iran with an area of 122 608 square kilometers.^10^ The information extracted and used in this study included age at death, year of death, and gender. Causes of death were coded using the 10th edition of the International Classification of Diseases (ICD-10). The relevant codes for cerebrovascular diseases were I60-I69.

 To assess the crude mortality rate due to cerebrovascular disease, we used the national census population from 1996 to 2016. We estimated the population based on annual growth for the remaining years. To eliminate or minimize the influence of age composition, standardized mortality rate was used. To calculate the age standardized mortality rate of cerebrovascular disease, we used the 2013 standard population for low- and intermediate-income countries.^11^

 The direct method of standardization involves the application of age-specific rates in a population of interest to a standard age distribution in order to eliminate differences in observed rates that result from differences in population composition.

 Compared to the Segi standard, the WHO standard has fewer children proportionally and a greater proportion of adults aged 70 + . To produce reliable indicators and better understand the age structure of low- and middle-income countries, a standard for these settings is necessary.^12^

 We use a standardized life table to calculate YLL and define life expectancy and the number of deaths due to cerebrovascular diseases for different sex and age groups. Calculation was made using the following equation^13^:


$$YLL=N\;Ce^{\left(ra\right)}/{\left(\beta +r\right)}^{2}\;\left[e^{-\left(\beta +r\right)\left(L+a\right)}\;\left[-\left(\beta +r\right)\left(L+a\right)-1\right]\;-e^{-\left(\beta +r\right)a}\;\left[-\left(\beta +r\right)\;a-1\right]\right]$$

N = the count of deaths for a certain age and gender.

 L = the rest of life expectancy at the age of death

 r = Discounting Rate equal to 0.03.

 β = the contract rate in calculating the age value equal to 0.04.

 C is a modified constant value equal to 0.1658, β is equal to 0.04, and these two numbers estimate the value of different ages (x).

 a = age at death

 e is fixed and equivalent to 2.71.

 The trends in YLL rate were analyzed using the joinpoint regression model, based on the log-linear model. Joinpoint regression analysis shows changing trends over successive segments of time and the number of increases or decreases within each part. The resulting line segment between joinpoints is defined by the annual percent change (APC), based on the slope of the line segment and the average annual percent change (AAPC). The analysis for the trend was performed using the Joinpoint Regression software 4.9.0.0.

 The Joinpoint regression model can be written as:


$$Log\left(yi\right)=\beta 0+\beta 1ti+Y1{\left(ti-Ti\right)}^{+}+...+YK{\left(ti-Tk\right)}^{+}+\varepsilon i,\;i=1,.......,n$$

Where ti indicates the time points (2004, 2005… 2019), yi represents the YLL rates, K shows the number of change points, β0, β1 and γ1… γk indicate the regression coefficients and εi is the model error term. By fitting the joinpoint regression, we can calculate the APC in rates between the estimated change points. To do this, the log transform of the model is utilized:


$$APC=100\times \left(\exp\;\left(\beta 1+Y1*I\;\left(ti-Ti\right)+...+Yk*I\;\left(ti-Tk\right)\right)-1\right)$$

.^14^

 The denominator of the fraction to calculate the YLL rate in each year was the population of each year, separately for men and women.

 The analysis of the count of YLL as a result of premature mortality caused by cerebrovascular diseases was performed using the YLL template of 2015, the WHO in the Excel spreader program version 2016.

 The protocol of the present study was reviewed and approved by the ethics committee of Shiraz University of Medical Sciences. All aspects of the study were conducted in compliance with the University Code of Ethics.

## Results

**Figure 1:**
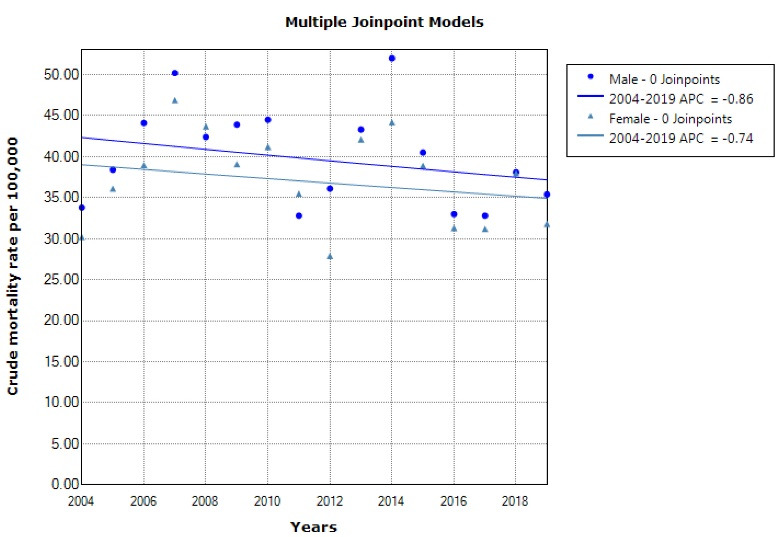
Trend of Crude Mortality Rate due to Cerebrovascular Diseases in Males and Females from 2004 to 2019.

**Figure 2:**
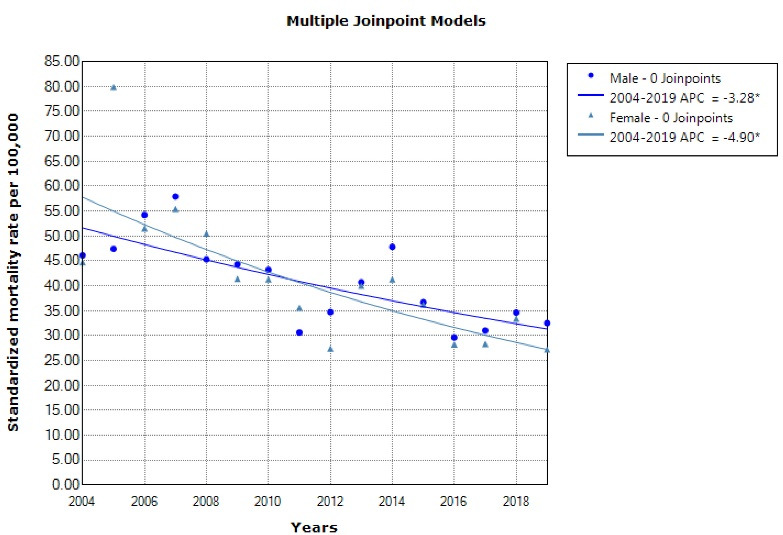
Trend of Standardized Mortality Rate due to Cerebrovascular Diseases in Males and Females from 2004 to 2019.

 From 2004 to 2019, a total of 24,051 deaths occurred due to cerebrovascular diseases in the Fars province with 12,586 cases in men (52.3%) and 11,465 cases in women (47.7%). The crude mortality rate in males increased from 33.8 in 100 000 people in 2004 to 35.4 in 100 000 people in 2019 (*P* = 0.298) while in females, it increased from 30.2 in 100 000 people in 2004 to 31.8 in 100 000 people in 2019 (*P* = 0.394) (Figure 1). The standardized mortality rate in males decreased from 46.1 in 100 000 population in 2004 to 32.5 in 100 000 population in 2019 (*P* = 0.001). In women, the standardized mortality rate decreased from 44.7 in 100 000 population in 2004 to 27.2 in 2019 (*P* < 0.001) (Table 1, Figure 2).

**Table 1:** Crude Mortality Rate and Age-standardized Mortality Rate (Per 100 000 Population) of Cerebrovascular Diseases Stratified by Gender and Year in Fars (Iran) 2004–2019

**Year**	**Number of Deaths**			**Crude Mortality Rate (Per 100000 Persons)**			**Age-Standardized Mortality Rate (95% CI)**		
	**Men**	**Women**	**Total**	**Men**	**Women**	**Total**	**Men**	**Women**	**Total**
2004	629	536	1165	33.8	30.2	32.0	46.1 (43.5-48.7)	44.7 (42.1-47.2)	45.4 (43.6-47.2)
2005	711	642	1353	38.4	36.1	37.3	47.4 (44.5-50.2)	49.9 (47.1-52.7)	48.8 (46.8-80.8)
2006	816	704	1520	44.1	39.0	41.6	54.2 (51.2-57.2)	51.5 (48.6-54.4)	52.8 (50.7-54.9)
2007	938	858	1796	50.2	46.9	48.6	57.9 (54.7-61.1)	55.4 (52.3-58.6)	56.6 (54.4-58.9)
2008	800	809	1609	42.4	43.7	43.0	45.3 (42.4-48.3)	50.5 (47.5-53.5)	47.9 (45.8-50.0)
2009	837	734	1571	43.9	39.1	41.6	44.3 (41.4-47.3)	41.4 (38.6-44.3)	42.9 (40.8-44.9)
2010	855	782	1637	44.5	41.2	42.8	43.2 (40.2-46.2)	41.3 (38.4-44.2)	42.1 (40.0-44.2)
2011	636	683	1319	32.8	35.5	34.1	30.6 (28.1-33.2)	35.6 (32.9-38.2)	33.0 (31.2-34.8)
2012	710	542	1252	36.1	27.9	32.0	34.7 (32.1-37.4)	27.4 (25.1-29.8)	31.0 (29.2-32.7)
2013	865	828	1693	43.3	42.1	42.7	40.7 (37.8-43.6)	40.0 (37.1-42.9)	40.2 (38.2-42.2)
2014	1052	878	1930	52.0	44.2	48.1	47.8 (44.6-50.9)	41.3 (38.4-44.2)	44.4 (42.3-46.6)
2015	829	781	1610	40.5	38.9	39.7	36.7 (34.0-39.5)	36.2 (33.4-38.9)	36.4 (34.5-38.3)
2016	686	634	1320	33.0	31.3	32.2	29.6 (27.2-32.1)	28.2 (25.7-30.6)	28.9 (27.1-30.6)
2017	682	633	1315	32.8	31.2	32.0	31.0 (28.5-33.4)	28.3 (25.9-30.7)	29.5 (27.8-31.2)
2018	796	772	1568	38.1	38.0	38.0	34.6 (31.9-37.2)	33.4 (30.7-36.1)	33.9 (32.0-35.8)
2019	744	649	1393	35.4	31.8	33.6	32.5 (29.9-35.0)	27.2(24.8-29.6)	29.7 (28.0-31.5)
Total	12586	11465	24051	40.0	37.3	38.6	41.9 (41.2-42.6)	39.2 (38.6-39.9)	40.5 (40.0-41.0)
*P* value	—	—	—	0.298	0.394	0.394	0.001	< 0.001	< 0.001

 All YLL to premature mortality from cerebrovascular disease during the 16-year study period were 119,436 (3.8 per 1000 persons) in men, 111,172 (3.6 per 1000 persons) in women, and 230,608 (3.7 per 1000 persons) in both sexes (Tables 2 and 3).

**Table 2:** Years of Life Lost Due to Cerebrovascular Diseases, Stratified by Gender, Age Groups and Year in Fars (Iran) 2004-2019

**Variables**		**2004**	**2005**	**2006**	**2007**	**2008**	**2009**	**2010**	**2011**	**2012**	**2013**	**2014**	**2015**	**2016**	**2017**	**2018**	**2019**	**Total**
		**No. YLL**	**No. YLL**	**No. YLL**	**No. YLL**	**No. YLL**	**No. YLL**	**No. YLL**	**No. YLL**	**No. YLL**	**No. YLL**	**No. YLL**	**No. YLL**	**No. YLL**	**No. YLL**	**No. YLL**	**No. YLL**	**No. YLL **
0-19	Women	59	86	59	29	116	86	57	88	479	58	211	269	88	177	61	89	2012
	Men	86	58	86	56	0	60	88	116	907	264	531	265	118	289	147	29	3100
20-34	Women	158	107	190	349	160	430	298	54	295	272	459	242	52	211	159	107	3543
	Men	288	286	448	212	343	237	396	188	1271	336	1401	654	212	315	104	342	7033
35-49	Women	439	729	530	621	411	420	453	204	700	469	503	466	345	343	605	278	7516
	Men	716	863	1048	795	595	834	641	404	988	597	1289	896	614	615	662	556	12113
50-64	Women	1243	1728	1611	1897	1969	1552	1301	1193	1150	1729	1543	1710	1339	1427	1265	1030	23687
	Men	1295	1587	1554	1986	1660	1967	1871	1393	2187	2034	2539	2036	1643	1726	1825	1751	29054
65-79	Women	3092	3222	3621	3877	3615	2817	2900	2764	1899	2854	3267	2895	1990	2140	2700	2398	46051
	Men	3484	3464	3630	3982	3100	2657	2772	1975	1802	2569	2828	2335	1914	2061	2135	2162	42870
+ 80	Women	912	1203	1383	1981	1879	1933	2133	1858	1293	2205	2217	2014	1773	1647	2145	1787	28363
	Men	706	1006	1327	1671	1638	1839	1903	1464	1239	1938	2168	1799	1529	1414	1925	1700	25266
Total	Women	5903	7075	7394	8754	8150	7238	7142	6161	5816	7587	8200	7596	5587	5945	6935	5689	111172
	Men	6575	7264	8093	8702	7336	7594	7671	5540	8394	7738	10756	7985	6030	6420	6798	6540	119436

**Table 3:** YLL Rate Per 1000 Person and YLL Trend by Gender and Age Groups in Fars (Iran) 2004-2019

**Age groups**	**YLL (y)**			**YLL Rate (Per 1000 Person)**			**AAPC for YLL Trend**			**P for YLL Trend**		
	**Men**	**Women**	**Total**	**Men**	**Women**	**Total**	**Men**	**Women**	**Total**	**Men**	**Women**	**Total**
0-19	3100	2012	5112	0.30	0.20	0.25	-2.7 (-26.0, 27.8)	6.0 (-4.0, 17.0)	1.8 (-22.3,33.4)	0.350	0.227	0.252
20-34	7033	3543	10576	0.71	0.36	0.53	-6.0 (-8.2, 7.7)	-2.2 (-9.6, 5.8)	-1.0 (-7.6,6.0)	0.877	0.551	0.750
35-49	12113	7516	19629	1.93	1.22	1.58	-4.2 (-7.3, -1.0)	-5.4 (-8.9, -1.8)	-4.6 (-7.7, -1.5)	0.014	0.007	0.007
50-64	29054	23687	52741	8.54	6.94	7.74	-3.4 (-5.0, -1.7)	-5.9 (-7.8, -4.0)	-4.5 (-6, -3.1)	0.001	< 0.001	< 0.001
65-79	42870	46051	88921	31.37	33.67	32.52	-4.9 (-6.6, -3.2)	-5.8 (-7.6, -4.0)	-5.3 (-7.0, -3.7)	< 0.001	< 0.001	< 0.001
+ 80	25266	28363	53629	59.22	74.08	66.25	-2.0 (-4.3, -0.3)	-2.1 (-4.1, -0.1)	-2.1 (-4.2, 0.1)	0.088	0.042	0.055
Total	119436	111172	230608	3.79	3.61	3.70	-1.6 (-3.4, 0.3)	-2.0 (-3.6, -0.4)	-1.8 (-3.4, -0.2)	0.098	0.017	0.032

 Among all age groups, cerebrovascular diseases had the highest and lowest YLL for both genders in the age group of 65–79 years and under 20 years, respectively (Table 2).

###  The Trend of YLL Caused by Cerebrovascular Diseases

**Figure 3:**
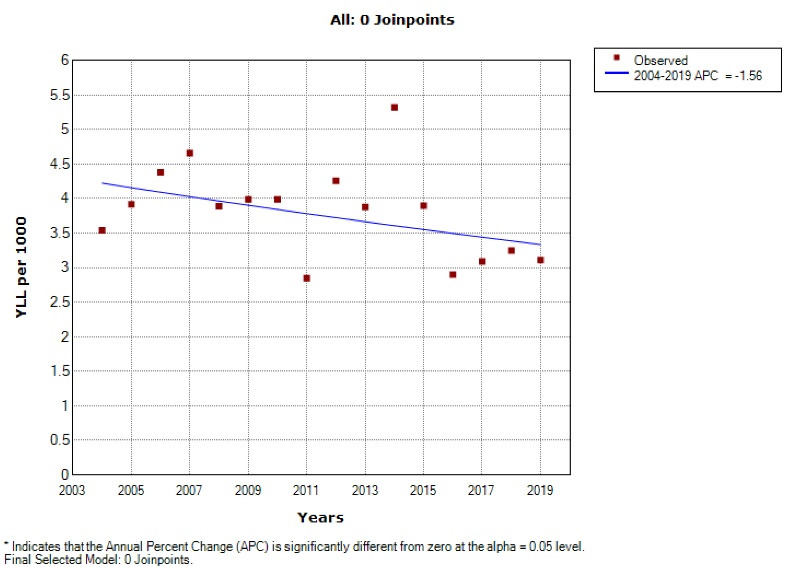
Trend of YLL due to Cerebrovascular Diseases in Males from 2004 to 2019.

**Figure 4:**
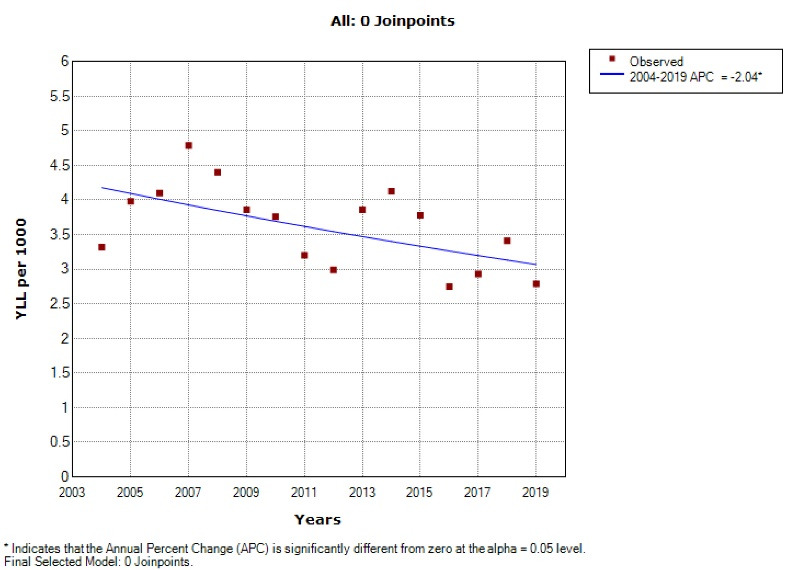
Trend of YLL due to Cerebrovascular Diseases in Females During 2004–2019.

 Based on the joinpoint regression, the 16-year trend of YLL rate due to premature death was decreasing: APC was -1.6% (95% CI -3.4 to 0.3, *P* = 0.098) for males, -2.0% (95% CI -3.6 to -0.4, *P* = 0.017) for females and -1.8% (95% CI -3.4 to -0.2, *P* = 0.032) for both sexes. The model did not show any joinpoint, and hence, the AAPC is the same as APC (Figures 3 and 4).

## Discussion

 Our study shows the mortality rate and YLL caused by cerebrovascular diseases in the Fars province (southern Iran) and its trend over the past 16 years. The main findings of our study showed that the standardized mortality rate from cerebrovascular diseases has decreased significantly from 2004 to 2019 in southern Iran. Cerebrovascular disease has been a major cause of death in most developed countries and has declined considerably in recent decades.^15^ According to studies, stroke mortality decreased in the second half of the twentieth century in North America and parts of Europe.^16^ In some countries, such as Japan, cerebrovascular mortality declined dramatically between 1960 and 2000. Studies have shown that hypertension and salt intake have been managed and reduced. Recently, however, in some areas, especially in Central Asia, mortality from cerebrovascular diseases has increased (slightly higher in men than women).^2,17,18^ Analysis of results from the European Union has shown that in some countries, mortality rate has increased among men while in others, it has risen among women.^19^

 Thus, one possible explanation for the reduction in stroke mortality in many countries may be related to the reduction or increase in stroke risk factors that lead to changes in disease incidence, as noted in other studies.^17^ The findings of our study showed that the reduction in standardized mortality rate due to cerebrovascular diseases is greater in women than men. However, in some studies, it has been shown that the decreasing trend of this rate in males is greater than in females, which contradicts the results of our study.^20,21^ However, in a study conducted in the Slovak Republic, the reduction in the standard rate of cerebrovascular mortality was the same in men and women, and in general, the mortality rate was greater in males than females.^22^ Reasons for changes in the trend may be due to changes in diagnostic and treatment methods, changes in disease registration, and better completion of a death certificate or a combination of these factors. However, studies conducted in Iran have shown that the trend of hypertension in Iran has been declining, which can also be a reason for this decrease.^23,24^ Although the rate of cerebrovascular mortality in our study is declining, the standardized mortality rate in 2019 was 32.5 in 100 000 population for men and 27.2 for women, compared to the study in European countries, where the standardized rate of cerebrovascular disease in men and women is about 80 and 100 in 100 000 population respectively. In this study, the standardized rate of cerebrovascular death in women was higher than men.^19^ Among Asian countries, Japan used to have one of the highest rates of cerebrovascular mortality in men (433 in 100 000 population) in 1950, but this rate dropped significantly to less than 100 in 100 000 in 2004. ^2,25^ In our study, the total number of YLL due to premature death from cerebrovascular disease decreased during the 16 years and was the same in both sexes. However, in a study conducted in northwestern Iran (Kurdistan), DALY was higher in men than women, and more than 95% of this DALY was related to YLL due to premature mortality, indicating a high mortality rate of cerebral stroke.^26^ Other studies have reported a reduction in YLL due to cerebrovascular disease.^27^ This declining trend may be due to several reasons, including improvements in medical treatment techniques.

 A jump in YLL was seen in 2014. We looked at deaths in different age groups. In 2014, the number of deaths in younger age groups was higher than others, which is one of the possible reasons for the high number of YLL in this year.

 For example, the number of deaths in people under the age of 70 during the years 2004 to 2019 was 211, 202, 233, 233, 195, 203, 198, 152, 294, 243, 349, 259, 187, 218, 207 and 214, respectively.

 Some limitations of this study include the possibility of undercounting the cases of death due to cerebrovascular diseases, whereas some of the strengths of the study are the wide period of time and the appropriate sample size. This study is one of the few studies which analyzes the trend of the YLL due to cerebrovascular diseases.

 In conclusion, the trend of mortality rate and YLL caused by cerebrovascular diseases has been decreasing in our study. Necessary measures, mainly primary and secondary prevention, should be taken to continue the diminishing trend of cerebrovascular diseases.
